# Supplementary material for: Designing a library of lived experience for mental health (LoLEM): protocol for integrating a realist synthesis and experience based codesign approach
Source: BMJ Open. 2023 Mar 8;13(3):e068548. doi: 10.1136/bmjopen-2022-068548 (PMC10008385; doi:10.1136/bmjopen-2022-068548)
Supplement: Author's manuscript [file bmjopen-2022-068548.draft_revisions.pdf]

BMJ Open

BMJ Open is committed to open peer review. As part of this commitment we make the peer review history of every article we publish publicly available.

When an article is published we post the peer reviewers' comments and the authors' responses online. We also post the versions of the paper that were used during peer review. These are the versions that the peer review comments apply to.

The versions of the paper that follow are the versions that were submitted during the peer review process. They are not the versions of record or the final published versions. They should not be cited or distributed as the published version of this manuscript.

BMJ Open is an open access journal and the full, final, typeset and author-corrected version of record of the manuscript is available on our site with no access controls, subscription charges or pay-per-view fees (http://bmjopen.bmj.com).

If you have any questions on BMJ Open's open peer review process please email
info.bmjopen@bmj.com

BMJ Open

Designing a Library of Lived Experience for Mental Health (LoLEM): Protocol for integrating a realist synthesis and Experience Based Co-Design approach

Journal:	*BMJ Open*
Manuscript ID	bmjopen-2022-068548
Article Type:	Protocol
Date Submitted by the Author:	28-Sep-2022
Complete List of Authors:	Lobban, Fiona; Lancaster University, Division of Health Research<br>Marshall, Paul; Lancaster University, Division of Health Research<br>Barbrook, John; Lancaster University, Lancaster University Library<br>Collins, Grace; n/a - Freelance participatory artist<br>Foster, Sheena; Lancaster University, Division of Health Research<br>Glossop, Zoe; Lancaster University, Division of Health Research<br>Inkster, Clare; Health Education England North West<br>Jebb, Paul; Lancashire and South Cumbria NHS Foundation Trust<br>Johnston, Rose; Lancaster University, Division of Health Research<br>Khan, Hameed; Lancaster University, Division of Health Research<br>Lodge, Christopher; Lancaster University, Division of Health Research<br>Machin, Karen; n/a - Independent survivor researcher<br>Michalak, Erin; The University of British Columbia<br>Powell, Sarah; Lancaster University, Lancaster Medical School<br>Rycroft-Malone, Jo; Lancaster University, Faculty of Health and Medicine<br>Slade, Mike; University of Nottingham School of Health Sciences; Nord University<br>Whittaker, Lesley; Lancashire and South Cumbria NHS Foundation Trust<br>Jones, Steven; Lancaster University, Division of Health Research
Keywords:	MENTAL HEALTH, PSYCHIATRY, QUALITATIVE RESEARCH

SCHOLARONE™
Manuscripts

Designing a Library of Lived Experience for Mental Health (LoLEM): Protocol for integrating a realist synthesis and Experience Based Co-Design approach

Authors –

Fiona Lobban
Division of Health Research, Lancaster University,
f.lobban@lancaster.ac.uk
corresponding author
ORCID iD: 0000-0001-6594-4350

Paul Marshall
Division of Health Research, Lancaster University,
p.marshall4@lancaster.ac.uk
ORCID iD: 0000-0002-3913-3359

John Barbrook
Lancaster University Library
j.barbrook@lancaster.ac.uk

Grace Collins
Freelance participatory artist
ge_collins@outlook.com

Sheena Foster
Division of Health Research, Lancaster University,
Sheenafoster@btinternet.com

Zoe Glossop
Division of Health Research, Lancaster University,
z.glossop@lancaster.ac.uk
ORCID iD: 0000-0002-6151-4860

Clare Inkster
Health Education England (North West)
clare.inkster@boltonft.nhs.uk

Paul Jebb
Lancashire and South Cumbria NHS Foundation Trust
Paul.jebb@lscft.nhs.uk

Rose Johnston
Division of Health Research, Lancaster University,
r.johnston2@lancaster.ac.uk
ORCID iD: 0000-0002-6863-2624

For peer review only

Hameed Khan
Division of Health Research, Lancaster University,
khan.hameed796@gmail.com

Chris Lodge
Division of Health Research, Lancaster University,
c.lodge@lancaster.ac.uk

Karen Machin
Independent survivor researcher
karen@role.org.uk
ORCID iD: 0000-0002-0374-4238

Erin E. Michalak
Department of Psychiatry
University of British Columbia, Vancouver, Canada
erin.michalak@ubc.ca
ORCID iD: 0000-0002-0812-6527

Sarah M. Powell
Lancaster Medical School, Lancaster University
s.m.powell@lancaster.ac.uk
ORCID iD: 0000-0002-3509-5789

Jo Rycroft-Malone
Faculty of health and Medicine, Lancaster University
j.rycroft-malone1@lancaster.ac.uk
ORCID iD: 0000-0003-3858-5625

Mike Slade,
School of Health Sciences, Institute of Mental Health, University of Nottingham, UK, AND, Nord University, Faculty of Nursing and Health Sciences, Health and Community Participation Division, Postbox 474, 7801 Namsos, Norway
M.Slade@nottingham.ac.uk
ORCID iD: 0000-0001-7020-3434

Lesley Whittaker
Lancashire and South Cumbria NHS Foundation trust
Lesley.Whittaker@lscft.nhs.uk

Steve Jones
Division of Health Research, Lancaster University,
s.jones7@lancaster.ac.uk
ORCID iD: 0000-0002-8801-5113

For peer review only

Corresponding Author
Fiona Lobban
Division of Health, Lancaster University
f.lobban@lancaster.ac.uk

Word count – 5533

For peer review only

Abstract

Introduction: People with lived expertise in managing mental health challenges can support other people facing similar challenges, and can advise carers how best to help. However, opportunities for sharing lived expertise are limited. Living libraries support people to be "living books", sharing experiences in dialogue with "readers" who can ask questions. Living libraries have been piloted world-wide in health-related contexts, but without a clear model of how they work or rigorous evaluation of their impacts. We aim to develop a programme theory about how a living library could improve mental health outcomes, and to co-design an implementation guide.

Methods and Analysis: We will use a novel integration of realist synthesis and Experience Based Co-Design (EBCD) to produce the programme theory and a guide to establishing a Library of Lived Experience for Mental Health (LoLEM). Two workstreams will run concurrently. (1) A realist synthesis of literature on living libraries will produce several programme theories, developed collaboratively with an Expert Advisory Group of stakeholders and through interviews. A systematic search will identify literature about living libraries. Data will be coded into our analysis framework, and we will use retroductive reasoning to explain living libraries' impacts. Individual interviews will refine and test theories. (2) Data from workstream 1 will inform ten EBCD workshops with people with experience managing mental health difficulties and health professionals, to produce a LoLEM implementation guide. Workstream 2 data will also inform the theory in workstream 1.

Ethics and Dissemination: Ethical approval was granted by Coventry and Warwick NHS Research Ethics Committee on 29th December 2021 (REF: 305975). The study outputs will be published as open access and shared through a knowledge exchange event, study website, mental health provider and peer support networks, peer-reviewed journals, and a funders report.

Registration Details: This protocol is registered on PROSPERO (CRD42022312789).

Strengths and limitations

- A Library of Lived Experience for Mental Health (LoLEM) has the potential to make a significant impact on improving mental health outcomes across public health, primary, and secondary mental healthcare, and training professionals including health and social care staff, police, and teachers.
- Consistent with the Medical Research Council (MRC) guidance for development of complex interventions, this novel methodology integrates a realist synthesis to develop a programme theory about what the likely impacts of a living library will be, for who, and in what context, with a series of Experience-Based Co-Design (EBCD) workshops. This will ensure the library is designed to be acceptable, engaging and feasible to implement across a range of settings, and will maximise positive and minimise negative impacts.
- The study design is creative, iterative, and involves relevant stakeholders, including people with lived expertise, in all aspects of design and delivery.

For peer review only

- The theory about how the living library will work to improve outcomes, and the utility of the implementation guide needs to be tested and refined in future research in which the LoLEM is delivered across a range of contexts.

Introduction

Approximately 1 in 6 adults and children in the UK experience mental health difficulties at any one time (1, 2), with similar rates reported around the world (3). Despite drug treatments and psychological interventions on offer, there is no evidence of these rates reducing. Indeed, following the impacts of the Covid-19 pandemic, rates are likely to increase (4). Health services are already unable to meet demand. New and inclusive ways to help society manage mental health are needed.

The value of learning to manage health from lived expertise is well recognised globally, pioneered by voluntary and third sector organisations. Value comes from the knowledge and support from people who have walked the same path, but also from the radically different relationship between the person sharing their expertise and those seeking help, which is one of mutual empowerment and growth, rather than professional to patient treatment (5).

Currently, there are several opportunities for people to share their lived expertise to help others. Around the world, committees addressing policy development, research funding and clinical guidelines (e.g. NICE guidelines in the UK) include people with lived experience. Training courses for mental healthcare professionals have lived experience reference groups contributing to curricula, and in mental health services, people are employed at a strategic level as peer leaders, members of patient/carer councils, and increasingly in the UK as Peer Support Workers (PSW), supported by growing evidence (e.g.(6)), and a competency framework and national training programme (7). However, evidence for the impacts of lived expertise support on patient outcomes is still limited (8, 9), and mechanisms of change are poorly understood. Further, all of these roles require significant investments of time, restricting them to people able to make this their job. This excludes the vast majority of people, and reduces the diversity of experience being shared.

More research is needed to develop and evaluate models of involvement that can facilitate wider involvement of those from diverse backgrounds (including people in work/with caring responsibilities). Here we examine one example; the living library model.

In a living library (also referred to as a human library), “readers” are people invited to select living “books” from a catalogue of synopses (provided by the books) and have a conversation. During this, they are encouraged to ask questions about things that matter to them. Hence, the library is not just about telling stories, but is about dialogue to inform, challenge our preconceptions and change the way we think. Books, readers and librarians have equal status, have the right to decline to answer any question, and may end the conversation if they wish. The living library concept originated in 2000 in Copenhagen and

For peer review only

has become the Human Library (10), originally face-to-face but now also online. Living libraries have since been used around the world across a range of health and non-health related contexts (11), but to our knowledge, are yet to be embedded in any sustained mental health support services.

A living library for mental health, offered as a sustained service, and shown to be effective, could overcome many limitations of existing involvement frameworks. People from diverse backgrounds or with seldom heard voices, could train to become a book, and flex their availability around other commitments, especially if the library was online and available beyond traditional working hours. Dialogue within the library enables readers from a range of backgrounds to explore directly the issues that they care about, and revisit conversations with books where appropriate. It allows the books to shape their stories and to share what they feel the reader needs to understand, which may be more effective than reading static written stories. Books can be trained and supported to create and share their stories in ways that feel safe and empowering for both books and readers. They remain in control of what is shared with who, how and when, and have direct experience of the immediate impacts of their sharing. Interaction face-to-face (or via video-link) may reduce our tendency to see people who have experienced mental health difficulties as different from us and "other", facilitating empathy and reducing stigma (12). Finally, the library metaphor may help engagement of books and readers, as libraries are familiar places, with a focus on enhancing wellbeing, e.g. (13), frequently attended (14), and positively perceived(15) by people experiencing mental distress.

The idea of a living library to improve mental health outcomes has international appeal and has already been tried around the world in a range of contexts including: improving self-management strategies for people diagnosed with bipolar disorder (16), reducing stigma in pharmacy students in Canada (17); and improving mental health literacy in students in Hong Kong (18). Living books have reported a wide range of benefits including; greater self-awareness (19), personal enjoyment and enhanced social relationships (20, 21), catharsis from sharing experiences (22), and feeling valued in promoting change(23). For readers, there is evidence for enhanced self-efficacy through discovering new ways to understand and manage specific difficulties (16). Direct contact for carers (formal and informal) with people with lived experience in an equal status context, can increase empathy, knowledge, perceived similarity, and reduce stigma and anxiety about contact (24).

However, living libraries also have the potential for negative impacts. All libraries are hosted within an organisational system, which in turns sits within a broader social context. Such contexts are never neutral, and can determine which stories are shared, how, and with what impacts (25). As has been shown with the use of recovery narratives in mental health services, even when the opportunity to share lived expertise stories is intended to be empowering, and promote diversity, the use of recovery stories in health and social contexts can serve to propagate normative organizational agendas through the selection and honing of stories to allow only those that are "acceptable" (26). Storytellers may fear unwanted consequences of sharing their experiences, such as loss of personal freedom,

For peer review only

especially if they have previously been treated under a section of the mental health act (27). The process of becoming a living book may be stressful, triggering of trauma experiences, or may confirm rather than challenge existing negative stereotypes if living libraries are not well designed and books are not given equal status to readers and library staff (28).

Our aim is to explore how a living library could be set up and used to train health and social care professionals, support people experiencing mental health difficulties and their carers, and reduce stigma in the general population across the UK. To do this, firstly we need to understand the likely impacts, both positive and negatives for books and readers, how these come about, for who, and in what context. Building this programme theory of how a living library is likely to work is a key early step in the MRC framework for the development of complex interventions (29) and ensures that we design the library within contexts that will successfully trigger key mechanisms of positive change, whilst reducing any potential harm. It also guides us to select ways of assessing important impacts (positive and negative) for both readers and books. Secondly, the library needs to be co-designed by a team of relevant stakeholders including people with lived expertise, mental health service providers, librarians, and commissioners. The processes of building the theory and co-designing the library protocol, must run in parallel so each can usefully inform the other. The evolving programme theory can identify contextual factors that need to be addressed in the co-design of the library. Reciprocally, issues raised by stakeholders during the co-design process can identify contextual factors that need to be theorised to understand how they are will impact on the success (or otherwise) of the library.

To meet our objectives, we will use a novel integration of a realist synthesis to build our programme theory, and a modified version of the Experience-Based Co-Design (EBCD) approach to developing the library protocol.

Realist synthesis is a theory-driven methodology that aims to explain how and why observed outcomes occur in different contexts (30). The approach is grounded in realist philosophy and proposes that complex interventions in health have intended and unintended impacts (outcomes-O) through the way in which people respond to the programme resources offered (mechanism-M). The extent to which these mechanisms are triggered depends on specific features of the environment in which the programme is delivered, or characteristics of the participants (context-C). Realist synthesis is a useful early step in developing theory-informed interventions in healthcare. However, the processes by which the theories developed and then used to inform the design process are often not well articulated. In this study we show how a realist synthesis can be run in parallel with a modified version of the EBCD approach to develop interventions which are both theory-informed, and fully grounded in lived experience.

EBCD is a qualitative approach which captures in-depth experiences of stakeholders, identifies emotionally charged points in the care process, and uses these as a focus for co-designing changes to care to improve outcomes (31). Positive impacts of the process have been reported by people taking part in the process within a mental health context (e.g. (32)). EBCD has largely been conducted with the intention of service improvement but has

For peer review only - http://bmjopen.bmj.com/site/about/guidelines.xhtml

been recognised as having potential for early-stage intervention design projects, when combined with programme theory development (33). For example, Fylan et al. provide a detailed description of how they integrated EBCD with the use of behaviour change theories and methods to develop and test interventions for safer medicines use across healthcare settings (34). However, we were unable to identify another study that combined EBCD with a realist synthesis process. Therefore, we have outlined our protocol in detail, and following PRISMA-P guidelines (35), in the hope this will be of use to others in developing other theory-informed interventions.

Aims and objectives

Our aim is to co-design a theory informed Library of Lived Experience for Mental Health (LoLEM)

Our objectives are:

1. To build a programme theory for the LoLEM using a realist synthesis of research and practice literature with primary data from interviews with people who have been involved in living libraries. Our theory will include likely positive and negative impacts of the LoLEM on books, readers, and librarians, and across a range of different contexts.
2. To co-design a guide for delivering a LoLEM. Through a series of co-design workshops with a multi-stakeholder group, and drawing on the developing theory, will design key features of the LoLEM, outlining core components needed to successfully deliver and evaluate a living library, and highlighting flexible adaptation to aid implementation and effectiveness across different contexts.

Our team includes people with experience in group facilitation and peer support (KM, CL), mental health professionals (PJ, LW), clinical academics (FL, SJ, JRM), information specialists (JB), researchers (PM, RJ, ZG) and an artist (GC). We have established a non-independent Expert Group to oversee and guide the project. This includes five people with expertise in hosting (EEM, CI, SP), researching (MS), or taking part in a living library (HK), and a carer with a broad interest in lived expertise frameworks (SF). The group will be consulted at each of the steps described below throughout the study, via monthly online video meetings.

Methods and analysis

Our study is underway and will run from the end of September 2021 to the end of January 2023. We will run two workstreams in parallel to ensure each informs the other. Weekly operational meetings for each stream will guide activity and facilitate synergy. Monthly meetings with the Expert Group will support strategic direction, problem-solving, and ensure this work is situated within the broader context of peer support in mental health. The study design is shown in Figure 1.

For peer review only

Add figure 1 here Figure 1. Library of Lived Experience for Mental Health (LoLEM) Study Design

Workstream 1- Realist Synthesis

The aim of our realist synthesis is to develop a programme theory about what the likely impacts would be of a LoLEM, for who, how, and in what context. Following established realist methodology (30, 36), we will develop our programme theory of how living libraries "work", drawing on relevant "middle-range" theories, reviewing relevant literature; and conducting in-depth interviews with key stakeholders. Key steps are described in detail below.

1. Refine the scope of the review.

The scope of our review was refined through early discussion with our Expert Group at the outset of the study. The aims were to: estimate the size of relevant literature, specify terms to include in our search strategy, interview stakeholders, and identify key papers for our search sensitivity analysis.

We undertook a broad scoping search of relevant electronic databases including: Web of Science, PsychINFO, Medline, SocINDEX and CINAHL, using search terms: "living librar*" OR "human librar*" OR "live librar*" OR "living book*" OR "human book*" OR "people book*" OR "live book*". We searched grey literature through Google/Google scholar (top 100 results), OpenGrey (multi-disciplinary), and ProQuest (theses & dissertations), using search terms "living library" OR "human library". We screened references and citations for key papers. We identified 44 published articles that reported an evaluation of a living library: 10 from a healthcare care context, (9 journal, 1 report) and the rest in non-health (educational) contexts. The majority of studies were small-scale qualitative evaluations of the impacts of temporary living libraries. There were many more reports of living libraries being run, but not evaluated. Findings showed that the living library concept has high face validity but more rigorous research is needed to test impacts and underlying mechanisms in different contexts.

Through discussion with our Expert Group, the scope of the review was set as in table 1.

Table 1: Scope of review

INTERVENTION	For the purpose of this review, living libraries (also referred to as human libraries) are defined as:<br>- Synchronous interaction between a human 'book' and 'reader'<br>- Conversations are time-limited and supported/hosted by 'librarians'<br>- Conversations relate to any aspect of psychosocial functioning/wellbeing<br>- Held under the name 'living library' or 'human library'
CONTEXT	Any living library in any setting. We will focus on any features of the library, or backdrop against which it is being delivered, or the

For peer review only

	individual members, that may influence how the books/readers/librarians respond to the library. This includes but is not limited to individual characteristics of people involved, organisational setup and location of the library, how books and readers are recruited, how books are trained and supported, and target population for readers
MECHANISM	The response of the books and readers to the resources offered by the library
OUTCOME	Any positive or negative impact on books or readers or librarians

Given the limited data available, we did not limit the scope to living libraries within a mental health context.

2. Developing initial programme theories

We will draw on lived experiences of the research team and Expert Group to develop our initial programme theories. We will first conduct individual interviews with members who have hosted a living library (CI), or been a human book (CL, HK), or a reader (FL, SJ), and refine emerging theories in group discussion.

We will generate initial propositional statements in the form of 'if… then' statements to begin to identify causal links between concepts(37), full Context – Mechanism – Outcome (CMO) statements, or "theory areas" i.e. broad concepts related to context, mechanism or outcomes, that are considered important.

3. Prioritisation of theory areas

Based on previous experience of this methodology, we anticipate generating a high number of initial programme theories and / or theory areas. With the Expert Group we will prioritise theories around which to build our analysis framework using NVivo (version 12)(38). The prioritization process will continue throughout the study. At this stage, we anticipate prioritising theories that: (1) are specific to the living library context, rather than theories which would be applicable to any peer-based intervention (2) attempt to explain both positive and negative possible negative impacts of the library, and (3) address pragmatic issues around implementation identified in EBCD workshops.

4. Identify relevant literature

Working with our Library information Specialist (JB), we will develop an initial search strategy that can be adapted for use across several electronic databases and can be refined iteratively as our theories develop.
Our first search will identify literature that can inform our theory about how the LoLEM might work. Given the limited literature available, we will include living libraries in any context, not specific to mental health. There are likely to be similarities (but also important differences) in underlying mechanisms of how living libraries work across contexts.

For peer review only

We will select multidisciplinary research databases in addition to relevant health, social science and education databases to maximise search coverage including: Web of Science, Scopus, PsychINFO, Medline, CINAHL, Embase, SocINDEX, Social Care Online, British Education Index, Education Abstracts, Library, and Information Science & Technology Abstracts.

Databases will be searched using the following terms: “living librar*” OR “human librar*”, applied in each database to ‘title’, ‘abstract’, and where available ‘keywords’ fields. Grey literature will be searched using: ProQuest (theses/conference documents), British Library via Ethos (theses), International Clinical Trials Registry Platform (clinical trials), Overton.io (UK health policy), Bielefeld Academic Search Engine (articles/these/conference documents), Google.com*

We will conduct google searches of the following domains for websites that included the words “human library” or “living library”. - Inurl: ac (academic institutions) - Inurl: edu (academic institutions) - Inurl: gov (government-published) - Inurl: org (non-governmental organisations) - Inurl: nhs (UK National Health Service).

We will test the sensitivity of the search strategy by ensuring it picks up five key papers identified from our literature scoping, in each of the electronic databases that includes the paper. We will include a wide range of data sources including primary data, reviews, commentaries, manuals, and detailed lived experience accounts. Inclusion and exclusion criteria are listed in table 2.

Table 2: Inclusion and exclusion criteria for living library systematic search.

**Inclusion criteria**
- Full-text available in English
- Published since 2000 (inception of the human library concept)
- Relates to human or living library (as per the above definition)
- Any study type is permitted
**Exclusion criteria**
- Non-written files (audio-visual content)

All identified documents will be uploaded to Endnote and duplicates removed. De-duplicated records will be uploaded into Rayyan (39) to facilitate a two-step screening process.

In step one, all documents will be screened by title and abstract with 20% screened by a second reviewer to check reliability. Any uncertainties will be discussed and refinements made to criterion descriptors as needed. At stage two, full texts will be read, firstly to check the inclusion criteria are met, and secondly to assess the additional criteria of rigour and relevance. Rigour will be assessed by the overall quality of the study or the theoretical arguments being put forward, and the confidence we have in the conclusions being drawn. Relevance will be assessed by the extent to which the document tells us anything useful to refine, refute, or elaborate our programme theories (40). We will not use formal assessment of methodological quality tools often used in systematic reviews. The value of individual studies to the development of the programme theory in a realist synthesis depends not only

For peer review only

on the methodological rigour, but also on what the document can tell us that is relevant to building our theory, and the conceptual thinking of the authors.

We will conduct hand searches of reference sections (backward searching) and citations (forward searching) of eligible documents included in the review after this full text screening stage. We will also consult members of our expert advisory group for any additional materials that may have been missed by our search strategy.

5. Data Extraction

Using a proforma designed for the study we will extract details about each included study including: publication details (authors, date, title); study details (location, design, sample characteristics); and intervention details (where applicable - format of the intervention). Reading the full paper in detail, and extracting data from all sections, we will extract any data that pertains to any of our identified theory areas, and code these extracts into our NVivo framework. We do not anticipate identifying full explicit CMO configurations, but will use inductive, deductive and abductive reasoning to work back from what is being said and infer what the underlying CMO configurations may be and code the data accordingly (41). Where data relevant to our theory is identified, but is not easily coded into our theory areas, we will refine, and add to our framework. At this point data from both workstreams will be integrated. Field notes and online interactive whiteboards created during the EBCD workshops will also be coded into the NVivo framework and will continue to inform the theory development throughout the rest of the study.

6. Data Analysis

A detailed analysis of each theory area will be done using retroductive reasoning. This involves working back from the data to identify the context–dependent mechanisms underlying the impacts described in the data that can help us to understand the impacts of living libraries across a range of different contexts, and understand why these impacts are occurring. Analysis will focus on refining, reconfiguring, elaborating, refuting, and consolidating each of the theory areas described in the NVivo framework. Here we will draw on several strategies including:

1- Comparing instances in which a living library has been used in very different contexts to understand how and why the outcomes are different.
2- Examining extreme cases where very positive or very negative outcomes are reported for a library or any specific individuals within the library
3- Examining unintended and unexpected outcomes to determine what contextual factors may have triggered mechanisms to generate these.
4- Using counterfactual thinking and thought experiments to consider whether a particular outcome could have occurred without a specific contextual factor being present, what is it about this contextual factor that leads to the outcome, and imagining how the outcome might have been different if a specific aspect of context was modified.

For peer review only

We hope this process will refine our initial programme theories, but we anticipate it will also generate further questions.

7. Draw on middle range theories

To further elaborate our theories and fill in any gaps in our CMO configurations, we will draw on middle-range theories that can explain how a LoLEM might work by theorising relevant mechanisms at a level of abstraction above the specifics of the living library model. For example, at the individual (micro) and interpersonal (meso) levels these may include Intergroup Contact Theory (42, 43), Social Identity Theory (44), Social Learning Theory (45), and Personal Recovery (46). Additional theories at the organisational (macro) level may also be relevant such as the theory of Health Empowerment (47). At this stage, and consistent with the iterative nature of realist synthesis methodology, we may conduct additional theory-driven literature searches for further middle range theories that augment the theory development process. The selection and use of middle range theories will be guided by the extent to which each theory helps us to further develop our programme theories.

8. Stakeholder interviews

Our second strategy to elaborate and refine our theories, will be to use realist interviewing (48) with key stakeholders. We will sample through snowballing techniques, initially drawing on the networks of our broad research team, Expert Group, and key individuals identified through our literature searching. Those willing to take part will be sent a participant information sheet and asked to consent online. Interviews will be done flexibly (to maximise participation) using individual/group interviews, online/face to face/telephone mediums, and topic guides designed to test our explanatory theories. Data will be recorded and transcribed, and coded and analysed within the theory framework in NVivo as described in step 5 & 6.

9. Synthesise final theories

In the final stage of the synthesis, we will use iterative discussion within our research team, and Expert Group, to consolidate our programme theories into a final framework that articulates how a LoLEM works, for whom and in what contexts. This will draw on data derived from literature searches, realist interviews, and from the EBCD workshops described below.

Workstream 2- Experience Based Co-Design (EBCD) of a LoLEM guide

Informed by the EBCD approach (49), we will adapt the process to meet the specific needs of this project. Key steps are outlined below

1. Gather experiences from stakeholders to understand key issues to be addressed

In EBCD these are referred to as “touchpoints” and are identified through interviews, observations, or group discussion. In this project, we will use the data from steps 1-3 of the realist synthesis to identify key features of living libraries that need to be co-designed.

For peer review only

2. Set up the co-design process

Participants will be recruited through NHS Trusts, peer support charities, and existing user involvement networks across the North of England to take part in a series of monthly co-design workshops. All participants will be sent a Participant Information Sheet and asked to consent to data from the workshops being used to inform study outputs. Participants will be paid in accordance with guidance from the National Institute of Health Research (50), and offered access to individual support from the research team. Workshops will be run online to maximise engagement geographically, and through pandemic restrictions. They will be co-facilitated by an experienced trainer in peer support interventions whose work is grounded in lived expertise (KM), our PPI lead for the project (CL), and an artist (GC) with experience in co-delivering and capturing co-design processes using creative visual tools. The process will be supported by other members of the research team.

3. Run the co-design workshops

The content of each workshop will be responsive to our developing programme theories and agreed in consultation with participants during the process. The initial plan consists of four phases to the process:

a. Building rapport and trust within the group through experiences of story sharing which are fundamental to the living library model

b. Applying the experiences of story sharing to generate further understanding of the living library model, and identify barriers and facilitators to success

c. Applying the understanding of the barriers and facilitators to develop fundamental guidelines and key considerations for running a living library in a range of settings

d. Agreeing format of production of the guidelines and next steps.

4. Generate a LoLEM guide for how to set up and run a LoLEM across a range of contexts.

The aim is not to write a “recipe book”, but to identify key things to consider when setting up a library, and to understand how different decisions are likely to lead to different outcomes. These will be described using text and artistic impressions. Key issues anticipated include: what the library is for, where and how the library is hosted, how books are recruited, trained, reimbursed, supported; who the readers are, and how the library is offered to them; how the role of the librarian is set up, and how a LoLEM should be evaluated.

5. Celebrate and share outputs

A celebration event will be held to share the final draft of the protocol and review the process of co-design.

For peer review only

Knowledge Mobilisation

Outputs from this study will include an expert informed theory of how a LoLEM could work to improve outcomes for books, and readers; and a theory informed co-designed protocol for setting up and running a LoLEM. These will be openly shared in open access journals, following RAMESES reporting guidelines (51) and through Lancaster University and Lancashire and South Cumbria NHS websites. Any amendments made to the protocol will be explained in the outcome paper on completion of the study, and updated on PROSPERO.

We also aim to have established a network of people with lived experience, and mental health support providers who are motivated to use the study outputs to establish a library. We will host a dissemination event to present the findings of the study to stakeholders including commissioners. We will invite mental health support providers, commissioners, health professional trainers, and other organisations who may benefit from hosting a LoLEM. We hope to use this to build a collaborative bid for further research funding to conduct a realist evaluation of a series of LoLEMs across different contexts.

Discussion

The value of lived experience in mental health support is well recognised, but more is needed to increase diversity in who can share their expertise, and how. Living libraries have been used in a range of health-related settings, but to our knowledge are yet to be offered as a sustainable part of services to support mental health. This study will provide a co-designed implementation guide about how a library of lived experience for mental health (LoLEM) could support people to manage their own mental health challenges, and support formal and informal carers to best care. Consistent with the MRC guidance on intervention design, and using a novel integration of realist synthesis with Experience Based Co-Design (EBCD), the guide will be theory driven, adaptable across a range of contexts, target key mechanisms of change, and identify important outcomes to assess.

Challenges

There are challenges to delivering this study. The first is ensuring the scope of the literature reviewed is broad enough to understand the key mechanisms driving the outcomes, whilst focussed enough to be feasible within the timeframe and resources of the project (funded for 1 year). Secondly, the timing of each workstream and communication between the two needs to be carefully managed so that they are mutually informative. We will address these challenges through regular consultation within the research team and with reference to a detailed Gantt chart.

Patient and Public Involvement statement

Lived expertise is embedded at every level of the project. During the study design, and prior to applying for funding, we held an online consultation with four service users from our Spectrum Centre Advisory Panel at Lancaster University (52), which informed the idea of a living library (two had been living books). Perceived benefits were that this approach can be engaging, non-stigmatising and draws on a familiar and non-threatening metaphor. It has the potential to involve a wide range of books and readers due to flexibility in

For peer review only

time/location/what is shared/ways of sharing, and the opportunity for in-depth dialogue. Challenges identified were the need for adequate training, supervision, and support for books, as well as ensuring empowerment, ownership and control by people with lived experience. Whilst designing the study, we hosted a stakeholder event (n = 8 online) with services users and mental health service staff across the region. Additional considerations identified in this process included: the need to accentuate the library as additional to (rather than alternative to) PSW roles, and the need to increase diversity and access to a broad range of living books across age, ethnicity, gender, sexuality, and types of experience.

The research team includes two PPI leads with lived experience (CL & KM). They contributed to the design of the study and application for funding. They are involved in strategic management meetings and practical delivery in both workstreams, including co-facilitating the EBCD workshops. They will be authors on all outputs and involved in dissemination including presentations, and as co-applicants for future grants.

Our Expert Group includes two people with lived expertise. One is a service user who has been a reader (or book) in a living library, and the other is a carer who has a lot of experience in service development at an advisory level.

Ethics and dissemination:

In the same way that a poorly designed LoLEM could be disempowering, stressful and even traumatizing for books, and could confirm rather than challenge existing stigma and prejudice, this could also be true for the process of co-designing the library guide. To mitigate against this, we will: conduct the study within a University (rather than NHS) context; ensure all EBCD workshops will be co-facilitated with people who have lived expertise; and ensure all participants will have access to one to one support from the research team throughout the process as required. EBCD group members will be purposively sampled to ensure a diverse range of views and minimize any power issues. We will encourage exploration of the role of a LoLEM within the broader social context of mental health support, as well as more detailed design features of the library.

All data will be provided with informed written consent and will be collected and stored securely. The detailed and personal nature of the EBCD workshop data makes it impossible to fully anonymise, so this data will not be shared openly. However, all outputs will be published in open access journals. Plain English summaries will be made available on the Lancaster University website.

Conclusion

The living library model offers an attractive opportunity to expand the range of ways in which people can share their lived expertise of mental health. This can support others facing challenges, educate formal and informal carers, and challenge stigma, prejudice and discrimination in society. This novel study design integrates a realist synthesis to develop a programme theory about the likely impacts of a LoLEM (for who, and in what context), with an EBCD approach, to design a library guide that is theory driven and can be adapted across

a range of contexts. Future collaborative research will be needed to pilot the library guide to test acceptability and feasibility in a range of settings, and to further test and refine the programme theory.

Authors contributions

FL is the guarantor. FL led the design, funding application, and will co-lead the running of the project. PM is an Early Career Researcher who led on the ethics application, and will lead on the realist synthesis and integration of theory between the workstreams. He will co-lead the management of the study. JB advised on the search strategy. GC will co-facilitate EBCD workshops and produce artistic impressions of the developing theory. ZG, PJ, RJ, LW will help with the running of the EBCD workshops, and RJ will also screen papers and extract data in the realist synthesis. CL and KM will co-facilitate the EBCD workshops, with KM leading on co-design process, and Cl on supporting public and patient involvement. JRM will provide methodological guidance and input into the realist synthesis. SJ will be organizational lead for the EBCD workstream and consult on theory development in the realist synthesis. SF, CI, HK, SP, EM, MS have all provided strategic guidance to the design of the study. All authors contributed to and approved the final version of all protocols.

Funding Statement

The study is funded by the National Institute of Health and Care Research (NIHR), Research for Patient Benefit (RfPB) Programme (NIHR203476). The views expressed are those of the authors and not necessarily those of the NIHR or the Department of Health and Social Care. The study is sponsored by Lancashire and South Cumbria NHS Foundation Trust. Mike Slade acknowledges the support of the NIHR Nottingham Biomedical Research Centre.

Competing Interests Statement

There are no competing interests to declare.

For peer review only

References

1. McManus S, Bebbington PE, Jenkins R, et al. Mental health and wellbeing in England: the adult psychiatric morbidity survey 2014: NHS digital; 2016.
2. NHS Digital. Mental Health of Children and Young People in England, 2020: Wave 1 follow up to the 2017 survey October 2020 2020 Available from: https://digital.nhs.uk/data-and-information/publications/statistical/mental-health-of-children-and-young-people-in-england/2020-wave-1-follow-up. Accessed September 2022
3. World Health Organization. World mental health report: transforming mental health for all: executive summary. World mental health report: transforming mental health for all: executive summary2022.
4. Wykes T, Bell A, Carr S, et al. Shared goals for mental health research: what, why and when for the 2020s. *J Ment Health* . 2021:1-9.
5. Basset T, Faulkner A, Repper J et al.Lived experience leading the way: Peer support in mental health. London: Together UK. 2010.
6. Gillard S, Bremner S, Patel A, et al. Peer support for discharge from inpatient mental health care versus care as usual in England (ENRICH): a parallel, two-group, individually randomised controlled trial. *Lancet Psychiatry.* 2022;9(2):125-36.
7. Health Education England. Supporting the individual's wellbeing, giving hope and supporting recovery 2021 Available from: https://www.hee.nhs.uk/our-work/mental-health/new-roles-mental-health/peer-support-workers. Accessed September 2022.
8. Chinman M, George P, Dougherty RH, et al. Peer support services for individuals with serious mental illnesses: assessing the evidence. Psychiatric Services. 2014;65(4):429-41.
9. Lloyd-Evans B, Mayo-Wilson E, Harrison B, et al. A systematic review and meta-analysis of randomised controlled trials of peer support for people with severe mental illness. *BMC Psychiatry.* 2014;14(1):39.
10. The Human Library Denmark [09/11/2019]. Available from: https://humanlibrary.org/. Accessed September 2022.
11. Little N, Nemutlu G, Magic J, et al. . Don't judge a book by its cover! The Living Library Organiser's Guide. 2011.
12. Corrigan PW, Morris SB, Michaels PJ, et al. Challenging the public stigma of mental illness: a meta-analysis of outcome studies. Psychiatric services. 2012;63(10):963-73.
13. The Reader [Available from: https://www.thereader.org.uk/.Accessed September 2022.
14. Taking Part 2015/16 quarter 2 statistical release. Department for Digital, Culture, Media & Sport London: DCMS; 2015 [Available from: https://www.gov.uk/government/statistics/taking-part-201516-quarter-2-statistical-release. Accessed September 2022.
15. Brewster L. The public library as therapeutic landscape: A qualitative case study. *Health & Place.* 2014;26:94-9.
16. Michalak EE, Morton E, Barnes SJ, et al.Supporting Self-Management in Bipolar Disorder: Mixed-Methods Knowledge Translation Study. *JMIR ment health.* 2019;6(4):e13493.
17. Patten SB, Remillard A, Phillips L, et al. Effectiveness of contact-based education for reducing mental illness-related stigma in pharmacy students. *BMC med educ.* 2012;12(1):1-9.
18. Chung EY-H, Tse TT-O. Effect of human library intervention on mental health literacy: a multigroup pretest-posttest study. *BMC Psychiatry*. 2022;22(1):73.
19. Kudo K, Motohashi Y, Enomoto Y, et al.Bridging differences through dialogue: Preliminary findings of the outcomes of the human library in a university setting. 2011.
20. Dobreski B, Huang Y. The joy of being a book: Benefits of participation in the human library. *Proc Assoc Inf Sci Technol*. 2016;53(1):1-3.
21. Frey C, Powell J. Building community in an academic library. Social Justice and Activism in Libraries: Essays on Diversity and Change. 2019.
22. Giesler MA. Humanizing Oppression: The Value of the Human Library Experience in Social Work Education. *J Soc Work Educ*. 2021.

For peer review only

23. Brown D, editor School libraries as power-houses of empathy: People for loan in The Human Library. IASL Annual Conference Proceedings; 2016.
24. Maunder RD, White FA. Intergroup contact and mental health stigma: A comparative effectiveness meta-analysis. *Clin Psychol Rev* 2019;72:101749.
25. Woods A, Hart A, Spandler H. The Recovery Narrative: Politics and Possibilities of a Genre. *Cult Med Psychiatry* 2022;46(2):221-47.
26. Yeo C, Rennick-Egglestone S, Armstrong V, , et al. Uses and Misuses of Recorded Mental Health Lived Experience Narratives in Healthcare and Community Settings: Systematic Review. *Schizophr Bull* 2021;48(1):134-44.
27. Llewellyn-Beardsley J, Rennick-Egglestone S, Pollock K, et al. 'Maybe I Shouldn't Talk': The Role of Power in the Telling of Mental Health Recovery Stories. *Qualitative Health Research.* 2022, p.10497323221118239.
28. Kwan CK. A Qualitative Inquiry into the Human Library Approach: Facilitating Social Inclusion and Promoting Recovery. *Int J Environ Res Public Health* 2020;17(9).
29. Skivington K, Matthews L, Simpson SA, , et al. Framework for the development and evaluation of complex interventions: gap analysis, workshop and consultation-informed update. 2021.
30. Pawson R, Greenhalgh T, Harvey G, et al. Realist synthesis: an introduction. ESRC Res Methods Program. 2004;2.
31. Bate P, Robert G. Experience-based design: from redesigning the system around the patient to co-designing services with the patient. *BMJ Qual Saf* 2006;15(5):307-10.
32. Springham N, Robert G. Experience based co-design reduces formal complaints on an acute mental health ward. *BMJ Open Qual* 2015;4(1):u209153. w3970.
33. Green T, Bonner A, Teleni L, et al. Use and reporting of experience-based codesign studies in the healthcare setting: a systematic review. *BMJ Qual Saf* 2020;29(1):64-76.
34. Fylan B, Tomlinson J, Raynor DK, et al.Using experience-based co-design with patients, carers and healthcare professionals to develop theory-based interventions for safer medicines use. *Res Social Adm Pharm* 2021;17(12):2127-35.
35. Shamseer L, Moher D, Clarke M, , et al. Preferred reporting items for systematic review and meta-analysis protocols (PRISMA-P) 2015: elaboration and explanation. *BMJ*. 2015;349.
36. Pawson R. The science of evaluation: a realist manifesto: Sage; 2013.
37. Pearson M, Brand SL, Quinn C,, et al. Using realist review to inform intervention development: methodological illustration and conceptual platform for collaborative care in offender mental health. *Implementation Science*. 2015;10(1):134.
38. QSR International Pty Ltd. Nvivo (Version 12) 2018. Available from: https://www.qsrinternational.com/nvivo-qualitative-data-analysis-software/home?_ga=2.181156967.1157585097.1662443725-1883585101.1657638724. Accessed September 2022.
39. Ouzzani M, Hammady H, Fedorowicz Z, et al. Rayyan—a web and mobile app for systematic reviews. *Syst Rev*. 2016;5(1):210.
40. Williams L, Rycroft-Malone J, Burton C, et al. Improving skills and care standards in the support workforce for older people: a realist synthesis of workforce development interventions. *BMJ open.* 2016;6(8):e011964.
41. Mukumbang FC, Kabongo EM, Eastwood JG. Examining the Application of Retroductive Theorizing in Realist-Informed Studies. *Int J Qual Methods*2021;20:16094069211053516.
42. Allport G. The Nature of Prejudice. Reading, MA.: Addison-Wesley; 1954 1979.
43. Pettigrew TF. Intergroup contact theory. *Annu Rev Psychol* 1998;49(1):65-85.
44. Tajfel H. Human groups and social categories: Studies in social psychology: CUP Archive; 1981.
45. Bandura A, Walters RH. Social learning theory: Prentice-hall Englewood Cliffs, NJ; 1977.

For peer review only

46. Leamy M, Bird V, Le Boutillier C, et al.Conceptual framework for personal recovery in mental health: systematic review and narrative synthesis. *Br J Psychiatry.* 2011;199(6):445-52.
47. Shearer NBC. Relationships of contextual and relational factors to health empowerment in women. *Res Theory Nurs Pract* 2004;18(4):357-70.
48. Manzano A. The craft of interviewing in realist evaluation. Evaluation. 2016;22(3):342-60.
49. The Kings Fund. EBCD: Experience-based co-design toolkit. 2013.
50. NIHR. Payment guidance for researchers and professionals. 2020.
51. Wong G, Greenhalgh T, Westhorp G, et al. RAMESES publication standards: realist syntheses. BMC medicine. 2013;11(1):21.
52. Spectrum Centre. Spectrum Centre - Getting Involved [Available from: https://www.lancaster.ac.uk/health-and-medicine/research/spectrum/research/getting-involved/#d.en.405692. Accessed September 2022.

For peer review only

Figure 1. Library of Lived Experience for Mental Health (LoLEM) Study Design

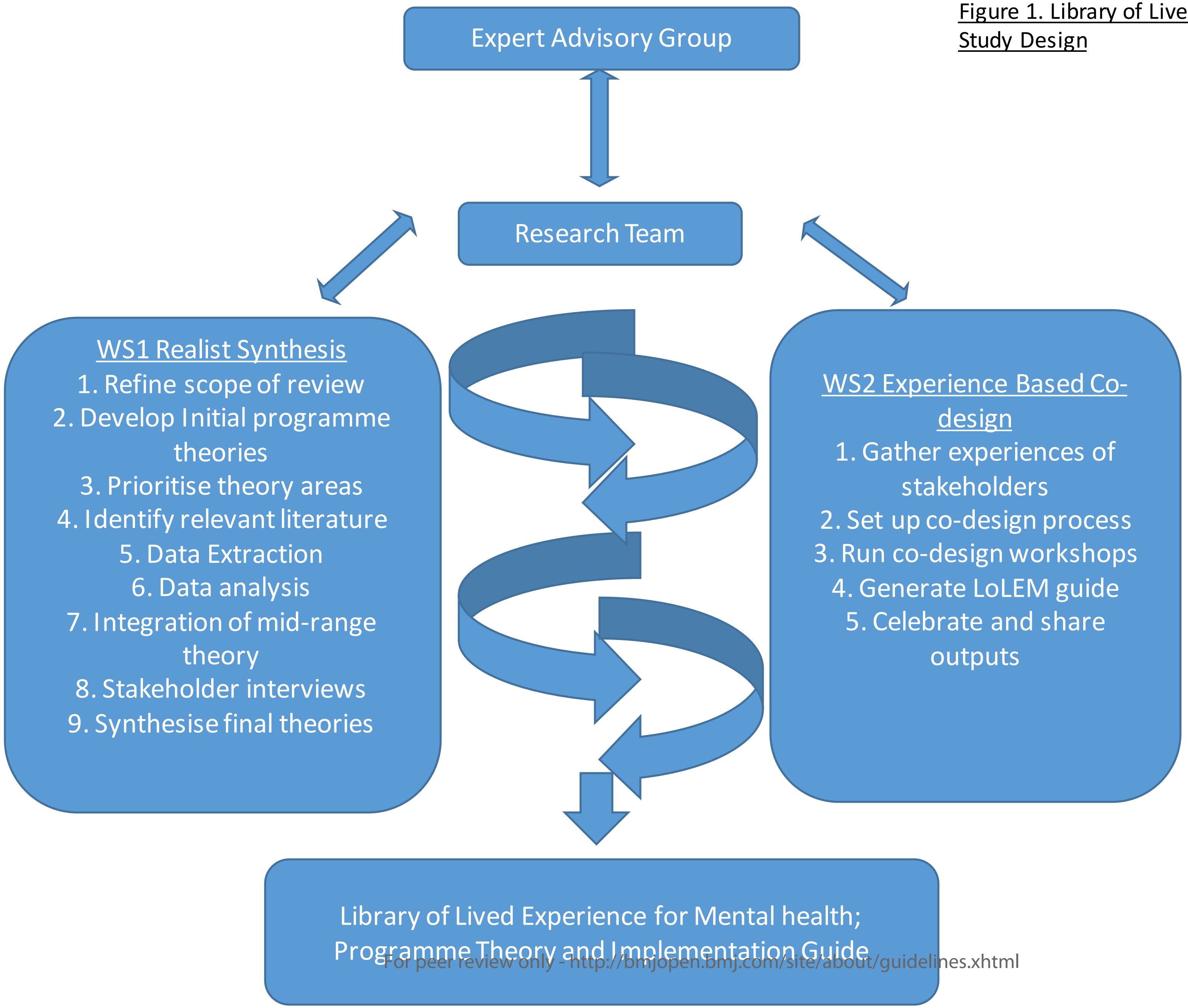

BMJ Open

Designing a Library of Lived Experience for Mental Health (LoLEM): Protocol for integrating a realist synthesis and Experience Based Co-Design approach

Journal:	*BMJ Open*
Manuscript ID	bmjopen-2022-068548.R1
Article Type:	Protocol
Date Submitted by the Author:	19-Jan-2023
Complete List of Authors:	Lobban, Fiona; Lancaster University, Division of Health Research<br>Marshall, Paul; Lancaster University, Division of Health Research<br>Barbrook, John; Lancaster University, Lancaster University Library<br>Collins, Grace; n/a - Freelance participatory artist<br>Foster, Sheena; Lancaster University, Division of Health Research<br>Glossop, Zoe; Lancaster University, Division of Health Research<br>Inkster, Clare; Health Education England North West<br>Jebb, Paul; Lancashire and South Cumbria NHS Foundation Trust<br>Johnston, Rose; Lancaster University, Division of Health Research<br>Khan, Hameed; Lancaster University, Division of Health Research<br>Lodge, Christopher; Lancaster University, Division of Health Research<br>Machin, Karen; n/a - Independent survivor researcher<br>Michalak, Erin; The University of British Columbia<br>Powell, Sarah; Lancaster University, Lancaster Medical School<br>Rycroft-Malone, Jo; Lancaster University, Faculty of Health and Medicine<br>Slade, Mike; University of Nottingham School of Health Sciences; Nord University<br>Whittaker, Lesley; Lancashire and South Cumbria NHS Foundation Trust<br>Jones, Steven; Lancaster University, Division of Health Research
**Primary Subject Heading**:	Mental health
Secondary Subject Heading:	Mental health
Keywords:	MENTAL HEALTH, PSYCHIATRY, QUALITATIVE RESEARCH

SCHOLARONE™
Manuscripts

Designing a Library of Lived Experience for Mental Health (LoLEM): Protocol for integrating a realist synthesis and Experience Based Co-Design approach

Authors –

Fiona Lobban
Division of Health Research, Lancaster University,
f.lobban@lancaster.ac.uk
corresponding author
ORCID iD: 0000-0001-6594-4350

Paul Marshall
Division of Health Research, Lancaster University,
p.marshall4@lancaster.ac.uk
ORCID iD: 0000-0002-3913-3359

John Barbrook
Lancaster University Library
j.barbrook@lancaster.ac.uk

Grace Collins
Freelance participatory artist
ge_collins@outlook.com

Sheena Foster
Division of Health Research, Lancaster University,
Sheenafoster@btinternet.com

Zoe Glossop
Division of Health Research, Lancaster University,
z.glossop@lancaster.ac.uk
ORCID iD: 0000-0002-6151-4860

Clare Inkster
Health Education England (North West)
Clare.Inkster@hee.nhs.uk

Paul Jebb
Lancashire and South Cumbria NHS Foundation Trust
Paul.jebb@lscft.nhs.uk

Rose Johnston
Division of Health Research, Lancaster University,
r.johnston2@lancaster.ac.uk
ORCID iD: 0000-0002-6863-2624

For peer review only

Hameed Khan
Division of Health Research, Lancaster University,
khan.hameed796@gmail.com

Chris Lodge
Division of Health Research, Lancaster University,
c.lodge@lancaster.ac.uk

Karen Machin
Independent survivor researcher
karen@role.org.uk
ORCID iD: 0000-0002-0374-4238

Erin E. Michalak
Department of Psychiatry
University of British Columbia, Vancouver, Canada
erin.michalak@ubc.ca
ORCID iD: 0000-0002-0812-6527

Sarah M. Powell
Lancaster Medical School, Lancaster University
s.m.powell@lancaster.ac.uk
ORCID iD: 0000-0002-3509-5789

Jo Rycroft-Malone
Faculty of health and Medicine, Lancaster University
j.rycroft-malone1@lancaster.ac.uk
ORCID iD: 0000-0003-3858-5625

Mike Slade,
School of Health Sciences, Institute of Mental Health, University of Nottingham, UK, AND, Nord University, Faculty of Nursing and Health Sciences, Health and Community Participation Division, Postbox 474, 7801 Namsos, Norway
M.Slade@nottingham.ac.uk
ORCID iD: 0000-0001-7020-3434

Lesley Whittaker
Lancashire and South Cumbria NHS Foundation trust
Lesley.Whittaker@lscft.nhs.uk

Steve Jones
Division of Health Research, Lancaster University,
s.jones7@lancaster.ac.uk
ORCID iD: 0000-0002-8801-5113

For peer review only

Sources of Support -

The study is funded by the National Institute of Health and Care Research (NIHR), Research for Patient Benefit (RfPB) Programme (NIHR203476). The views expressed are those of the authors and not necessarily those of the NIHR or the Department of Health and Social Care. The study is sponsored by Lancashire and South Cumbria NHS Foundation Trust. Mike Slade acknowledges the support of the NIHR Nottingham Biomedical Research Centre.

Competing interests statement

There are no competing interests to declare.

Word count – 5533

For peer review only

Abstract

Introduction: People with lived expertise in managing mental health challenges can be an important source of knowledge and support for other people facing similar challenges, and for carers to learn how best to help. However, opportunities for sharing lived expertise are limited. Living libraries support people with lived expertise to be "living books", sharing their experiences in dialogue with "readers" who can ask questions. Living libraries have been piloted world-wide in health-related contexts, but without a clear model of how they work or rigorous evaluation of their impacts. We aim to develop a programme theory about how a living library could be used to improve mental health outcomes, using this theory to co-design an implementation guide that can be evaluated across different contexts.

Methods and Analysis: We will use a novel integration of realist synthesis and Experience Based Co-Design (EBCD) to produce a programme theory about how living libraries work, and a theory and experience informed guide to establishing a Library of Lived Experience for Mental Health (LoLEM). Two workstreams will run concurrently. (1) A realist synthesis of literature on living libraries, combined with stakeholder interviews, will produce several programme theories. Theories will be developed collaboratively with an Expert Advisory Group of stakeholders who have hosted or taken part in a living library, and will form our initial analysis framework. A systematic search will identify literature about living libraries. Data will be coded into our analysis framework, and we will use retroductive reasoning to explain living libraries' impacts across multiple contexts. Individual stakeholder interviews will help refine and test theories. (2) Data from workstream 1 will inform ten EBCD workshops with people with experience of managing mental health difficulties and health professionals, to produce a LoLEM implementation guide. Data from this process will also inform the theory in workstream 1.

Ethics and dissemination: Ethical approval was granted by Coventry and Warwick National Health Service (NHS) Research Ethics Committee (REC) on 29th December 2021 (REF: 305975). The programme theory and implementation guide will be published as open access and shared widely through a knowledge exchange event, a study website, mental health provider and peer support networks, peer reviewed journals, and a funders report.

Registration details: This protocol is registered on PROSPERO (CRD42022312789).

Strengths and limitations

- Consistent with the Medical Research Council (MRC) guidance for development of complex interventions, this protocol describes a novel integration of a realist synthesis to develop a programme theory, with a series of Experience-Based Co-Design (EBCD) workshops to develop an implementation guide for a living library.
- The study design is creative, iterative, and involves relevant stakeholders, including people with lived expertise, in all aspects of design and delivery.
- The theory about how the living library will work to improve outcomes, and the utility of the implementation guide both need to be tested and refined in future research in which the LoLEM is delivered across a range of contexts.

For peer review only

Introduction

Approximately 1 in 6 adults and children in the UK experience mental health difficulties at any one time (1, 2), with similar rates reported around the world (3). Despite drug treatments and psychological interventions on offer, there is no evidence of these rates reducing. Indeed, following the impacts of the Covid-19 pandemic, rates are likely to increase (4). Health services are already unable to meet demand. New and inclusive ways to help society manage mental health are needed.

The value of learning to manage health from lived expertise is well recognised globally, pioneered by voluntary and third sector organisations. Value comes from the knowledge and support from people who have walked the same path, but also from the radically different relationship between the person sharing their expertise and those seeking help, which is one of mutual empowerment and growth, rather than professional to patient treatment (5).

Currently, there are several opportunities for people to share their lived expertise to help others. Around the world, committees addressing policy development, research funding and clinical guidelines (e.g. NICE guidelines in the UK) include people with lived experience. Training courses for mental healthcare professionals have lived experience reference groups contributing to curricula, and in mental health services, people are employed at a strategic level as peer leaders, members of patient/carer councils, and increasingly in the UK as Peer Support Workers (PSW), supported by growing evidence (e.g.(6)), and a competency framework and national training programme (7). However, evidence for the impacts of lived expertise support on patient outcomes is still limited (8, 9), and mechanisms of change are poorly understood. Further, all of these roles require significant investments of time, restricting them to people able to make this their job. This excludes the vast majority of people, and reduces the diversity of experience being shared.

More research is needed to develop and evaluate models of involvement that can facilitate wider participation of those from diverse backgrounds (including people in work/with caring responsibilities). Here we examine one example; the living library model.

In a living library (also referred to as a human library), “readers” are people invited to select living “books” from a catalogue of synopses (provided by the books) and have a conversation. During this, they are encouraged to ask questions about things that matter to them. Hence, the library is not just about telling stories, but is about dialogue to inform, challenge our preconceptions, and change the way we think. Books, readers and librarians have equal status, have the right to decline to answer any question, and may end the conversation if they wish. The living library concept is well established in the Human Library (10), originally face-to-face in Copenhagen, but now also online. However, living libraries have been used around the world across a range of health and non-health related contexts

(11), but to our knowledge, are yet to be embedded in any sustained mental health support services.

A living library for mental health, offered as a sustained service, and shown to be effective, could overcome many limitations of existing involvement frameworks. People from diverse backgrounds or with seldom heard voices could train to become a book, and flex their availability around other commitments, especially if the library was online and available beyond traditional working hours. Dialogue within the library enables readers from a range of backgrounds to explore directly the issues that they care about, and revisit conversations with books where appropriate. It allows the books to shape their stories and to share what they feel the reader needs to understand, which may be more effective than reading static written stories. Books can be trained and supported to create and share their stories in ways that feel safe and empowering for both books and readers. They remain in control of what is shared with who, how, and when, and have direct experience of the immediate impacts of their sharing. Interacting face-to-face (or via video-link) may reduce our tendency to see people who have experienced mental health difficulties as different from us and as “others”, facilitating empathy and reducing stigma (12). Finally, the library metaphor may help engagement of books and readers, as libraries are familiar places, with a focus on enhancing wellbeing, e.g. (13), frequently attended (14), and positively perceived (15) by people experiencing mental distress.

The idea of a living library to improve mental health outcomes has international appeal and has already been tried around the world in a range of contexts including: improving self-management strategies for people diagnosed with bipolar disorder (16), reducing stigma in pharmacy students in Canada (17); and improving mental health literacy in students in Hong Kong (18). Living books have reported a wide range of benefits including; a greater self-awareness (19), personal enjoyment and enhanced social relationships (20, 21), catharsis from sharing experiences (22), and feeling valued in promoting change (23). For readers, there is evidence for enhanced self-efficacy through discovering new ways to understand and manage specific difficulties (16). Direct contact for carers (formal and informal) with people with lived experience in an equal status context, can increase empathy, knowledge, perceived similarity, and reduce stigma and anxiety about contact (24).

However, living libraries also have the potential for negative impacts. All libraries are hosted within an organisational system, which in turns sits within a broader social context. Such contexts are never neutral, and can determine which stories are shared, how, and with what impacts (25). As has been shown with the use of recovery narratives in mental health services, even when the opportunity to share lived expertise stories is intended to be empowering, and promote diversity, the use of recovery stories in health and social contexts can serve to propagate normative organizational agendas through the selection and honing of stories to allow only those that are “acceptable” (26). Storytellers may fear unwanted consequences of sharing their experiences, such as loss of personal freedom, especially if they have previously been treated under a section of the mental health act (27). The process of becoming a living book may be stressful, triggering of traumatic experiences,

For peer review only

or may confirm, rather than challenge existing negative stereotypes if living libraries are not well designed and books are not given equal status to readers and library staff (28).

Our aim is to explore how a living library could be set up and used to train health and social care professionals, support people experiencing mental health difficulties and their carers, and reduce stigma in the general population across the UK. To do this, firstly we need to understand the likely impacts, both positive and negatives for books and readers, how these come about, for who, and in what context. Building this programme theory of how a living library is likely to work is a key early step in the MRC framework for the development of complex interventions (29) and ensures that we design the library within contexts that will successfully trigger key mechanisms of positive change, whilst reducing any potential harm. It also guides us to select ways of assessing important impacts (positive and negative) for both readers and books. Secondly, the library needs to be co-designed by a team of relevant stakeholders including people with lived expertise, mental health service providers, librarians, and commissioners. The processes of building the theory and co-designing the library protocol, must run in parallel so each can usefully inform the other. The evolving programme theory can identify contextual factors that need to be addressed in the co-design of the library. Reciprocally, issues raised by stakeholders during the co-design process can identify contextual factors that need to be theorised to understand how they will have impact on the success (or otherwise) of the library.

To meet our objectives, we will use a novel integration of a realist synthesis to build our programme theory, and a modified version of the Experience-Based Co-Design (EBCD) approach to develop the library protocol.

Realist synthesis is a theory-driven methodology that aims to explain how and why observed outcomes occur in different contexts (30). The approach is grounded in realist philosophy and proposes that complex interventions in health have intended and unintended impacts (outcomes-O) through the ways in which people respond to the programme resources offered (mechanisms-M). The extent to which these mechanisms are triggered depends on specific features of the environment in which the programme is delivered, or characteristics of the participants (contexts-C). Realist synthesis is a useful early step in developing theory-informed interventions in healthcare. However, the processes by which the theories are developed and then used to inform the design process are often not well articulated. In this study we show how a realist synthesis can be run in parallel with a modified version of the EBCD approach to develop interventions which are both theory-informed, and fully grounded in lived experience.

EBCD is a qualitative approach which captures in-depth experiences of stakeholders, identifies emotionally charged points in the care process, and uses these as a focus for co-designing changes to care to improve outcomes (31). Positive impacts of the process have been reported by people taking part in the process within a mental health context (e.g. (32)). EBCD has largely been conducted with the intention of service improvement but has been recognised as having potential for early-stage intervention design projects, when combined with programme theory development (33). For example, Fylan et al. provide a

For peer review only

detailed description of how they integrated EBCD with the use of behaviour change theories and methods to develop and test interventions for safer medicines use across healthcare settings (34). However, we were unable to identify another study that combined EBCD with a realist synthesis process. Therefore, we have outlined our protocol in detail, and followed PRISMA-P guidelines (35), in the hope this will be of use to others in developing other theory-informed interventions.

Aims and objectives

Our aim is to co-design a theory informed Library of Lived Experience for Mental Health (LoLEM)

Our objectives are:

1. To build a programme theory for the LoLEM using a realist synthesis of research and practice literature with primary data from interviews with people who have been involved in living libraries. Our theory will include likely positive and negative impacts of the LoLEM on books, and readers, across a range of different contexts.
2. To co-design a guide for delivering a LoLEM. Through a series of co-design workshops with a multi-stakeholder group, and drawing on the developing theory, will design key features of the LoLEM, outlining core components needed to successfully deliver and evaluate a living library, and highlighting flexible adaptation to aid implementation and effectiveness across different contexts.

Our Research Questions are:

1. What are the impacts of a LoLEM, on books and readers? How do these impacts happen, for whom, and in what contexts?

2. What are the key aspects to consider in designing a LoLEM?

Our team includes people with experience in group facilitation and peer support for mental health (KM, CL), mental health professionals (PJ, LW), clinical academics (FL, SJ, JRM), information specialists (JB), researchers (PM, RJ, ZG) and an artist (GC). We have established a non-independent Expert Group to oversee and guide the project. This includes five people with expertise in hosting (EEM, CI, SP), researching (MS), or taking part in a living library as a book with experience in managing mental health challenges (HK), and a mental health carer with a broad interest in lived expertise frameworks (SF). The group will be consulted at each of the steps described below throughout the study, via monthly online video meetings.

Methods and analysis

Our study is underway and will run from the end of September 2021 to the end of January 2023. We will run two workstreams in parallel to ensure each informs the other. Weekly operational meetings for each stream will guide activity and facilitate synergy. Monthly

meetings with the Expert Group will support strategic direction, problem-solving, and ensure this work is situated within the broader context of peer support in mental health. The study design is shown in Figure 1.

Add figure 1 here

Figure 1. Library of Lived Experience for Mental Health (LoLEM) Study Design

Workstream 1- Realist Synthesis

The aim of our realist synthesis is to develop a programme theory about what the likely impacts would be of a LoLEM, for who, how, and in what context. Following established realist methodology (30, 36), we will develop our programme theory of how living libraries "work", drawing on relevant "middle-range" theories, reviewing relevant literature; and conducting in-depth interviews with key stakeholders. Key steps are described in detail below.

1. Refine the scope of the review

The scope of our review was refined through early discussion with our Expert Group at the outset of the study. The aims were to: estimate the size of relevant literature, specify terms to include in our search strategy, interview stakeholders, and identify key papers for our search sensitivity analysis.

We undertook a broad scoping search of relevant electronic databases including: Web of Science, PsycINFO, Medline, SocINDEX and CINAHL, using search terms: "living librar*" OR "human librar*" OR "live librar*" OR "living book*" OR "human book*" OR "people book*" OR "live book*". We searched grey literature through Google/Google scholar (top 100 results), OpenGrey (multi-disciplinary), and ProQuest (theses & dissertations), using search terms "living library" OR "human library". We screened references and citations for key papers. We identified 44 published articles that reported an evaluation of a living library: 10 from a healthcare care context, (9 journals, 1 report) and the rest in non-health (educational) contexts. The majority of studies were small-scale qualitative evaluations of the impacts of temporary living libraries. There were many more reports of living libraries being run, but not evaluated. Findings showed that the living library concept has high face validity but more rigorous research is needed to test impacts and underlying mechanisms in different contexts.

Through discussion with our Expert Group, the scope of the review was set as in table 1.

Table 1: Scope of review

INTERVENTION	For the purpose of this review, living libraries (also referred to as human libraries) are defined as:

For peer review only

	- Synchronous interaction between a human ‘book’ and ‘reader’<br>- Conversations are time-limited and supported/hosted by ‘librarians’<br>- Conversations relate to any aspect of psychosocial functioning/wellbeing<br>- Held under the name ‘living library’ or ‘human library’
CONTEXT	Any living library in any setting. We will focus on any features of the library, or backdrop against which it is being delivered, or the individual members, that may influence how the books/readers/librarians respond to the library. This includes but is not limited to individual characteristics of people involved, organisational setup and location of the library, how books and readers are recruited, how books are trained and supported, and target population for readers
MECHANISM	The response of the books and readers to the resources offered by the library
OUTCOME	Any positive or negative impact on books or readers or librarians

Given the limited data available, we did not limit the scope to living libraries within a mental health context.

2. Developing initial programme theories

We will draw on lived experiences of the research team and Expert Group to develop our initial programme theories. We will first conduct individual interviews with members who have hosted a living library (CI), or been a human book (CL, HK), or a reader (FL, SJ), and refine emerging theories in group discussion.

We will generate initial propositional statements in the form of ‘if… then’ statements to begin to identify causal links between concepts(37), full Context – Mechanism – Outcome (CMO) statements, or “theory areas” i.e. broad concepts related to context, mechanism or outcomes, that are considered important.

3. Prioritisation of theory areas

Based on previous experience of this methodology, we anticipate generating a high number of initial programme theories and / or theory areas. With the Expert Group we will prioritise theories around which to build our analysis framework using NVivo (version 12) (38). The prioritization process will continue throughout the study. At this stage, we anticipate prioritising theories that: (1) are specific to the living library context, rather than theories which would be applicable to any peer-based intervention, (2) attempt to explain both positive and negative impacts of the library, and (3) address pragmatic issues around implementation identified in EBCD workshops.

4. Identify relevant literature

For peer review only

Working with our Library information Specialist (JB), we will develop an initial search strategy that can be adapted for use across several electronic databases and can be refined iteratively as our theories develop.
Our first search will identify literature that can inform our theory about how the LoLEM might work. Given the limited literature available, we will include living libraries in any context, not specific to mental health. There are likely to be similarities (but also important differences) in underlying mechanisms of how living libraries work across contexts.

We will select multidisciplinary research databases in addition to relevant health, social science and education databases to maximise search coverage including: Web of Science, Scopus, PsycINFO, Medline, CINAHL, Embase, SocINDEX, Social Care Online, British Education Index, Education Abstracts, Library, and Information Science & Technology Abstracts.
Databases will be searched using the following terms: "living librar*" OR "human librar*", applied in each database to 'title', 'abstract', and where available 'keywords' fields. Grey literature will be searched using: ProQuest (theses/conference documents), British Library via Ethos (theses), International Clinical Trials Registry Platform (clinical trials), Overton.io (UK health policy), Bielefeld Academic Search Engine (articles/these/conference documents), Google.com*
We will conduct google searches of the following domains for websites that included the words "human library" or "living library". - Inurl: ac (academic institutions) - Inurl: edu (academic institutions) - Inurl: gov (government-published) - Inurl: org (non-governmental organisations) - Inurl: nhs (UK National Health Service).

We will test the sensitivity of the search strategy by ensuring it picks up five key papers identified from our literature scoping, in each of the electronic databases that includes the paper. We will include a wide range of data sources including primary data, reviews, commentaries, manuals, and detailed lived experience accounts. Inclusion and exclusion criteria are listed in table 2.

Table 2: Inclusion criteria for living library systematic search screening.

**Inclusion criteria**
- Full-text available in English
- Published since 2000 (inception of the human library concept)
- Relates to human or living library (as per the above definition)
- Any study type is permitted
-Written text only (excluding audio-visual content)

All identified documents will be uploaded to Endnote and duplicates removed. De-duplicated records will be uploaded into Rayyan (39) to facilitate a two-step screening process.

In step one, all documents will be screened by title and abstract with 20% screened by a second reviewer to check reliability. Any uncertainties will be discussed and refinements made to criterion descriptors as needed.

At stage two, full texts will be read, firstly to check the inclusion criteria are met, and secondly to assess the additional criteria of rigour and relevance. Rigour will be assessed by the overall quality of the study or the theoretical arguments being put forward, and the confidence we have in the conclusions being drawn. Relevance will be assessed by the extent to which the document tells us anything useful to refine, refute, or elaborate our programme theories (40). Only studies considered to be relevant and of sufficient rigour for us to trust the implications for our theory development will be included. We will not use formal assessment of methodological quality tools often used in systematic reviews. The value of individual studies to the development of the programme theory in a realist synthesis depends not only on the methodological rigour, but also on what the document can tell us that is relevant to building our theory, and the conceptual thinking of the authors.

We will conduct hand searches of reference sections (backward searching) and citations (forward searching) of eligible documents included in the review after this full text screening stage. We will also consult members of our expert advisory group for any additional materials that may have been missed by our search strategy.

5. Data Extraction

Using a proforma designed for the study we will extract details about each included study including: publication details (authors, date, title); study details (location, design, sample characteristics); and intervention details (where applicable - format of the intervention). Reading the full paper in detail, and extracting data from all sections, we will extract any data that pertains to any of our identified theory areas, and code these extracts into our NVivo framework. We do not anticipate identifying full explicit CMO configurations, but will use inductive, deductive and abductive reasoning to work back from what is being said and infer what the underlying CMO configurations may be and code the data accordingly (41). Where data relevant to our theory is identified, but is not easily coded into our theory areas, we will refine, and add to our framework. At this point data from both workstreams will be integrated. Field notes and online interactive whiteboards created during the EBCD workshops will also be coded into the NVivo framework and will continue to inform the theory development throughout the rest of the study.

6. Data Analysis

A detailed analysis of each theory area will be done using retroductive reasoning. This involves working back from the data to identify the context–dependent mechanisms underlying the impacts described in the data that can help us to understand the impacts of living libraries across a range of different contexts, and understand why these impacts are occurring. Analysis will focus on refining, reconfiguring, elaborating, refuting, and consolidating each of the theory areas described in the NVivo framework. Here we will draw on several strategies including:

For peer review only

1- Comparing instances in which a living library has been used in very different contexts to understand how and why the outcomes are different.
2- Examining extreme cases where very positive or very negative outcomes are reported for a library or any specific individuals within the library.
3- Examining unintended and unexpected outcomes to determine what contextual factors may have triggered mechanisms to generate these.
4- Using counterfactual thinking and thought experiments to consider whether a particular outcome could have occurred without a specific contextual factor being present, what it is about this contextual factor that leads to the outcome, and imagining how the outcome might have been different if a specific aspect of context was modified.

We hope this process will refine our initial programme theories, but we anticipate it will also generate further questions.

7. Draw on formal theories

To further elaborate our theories and fill in any gaps in our CMO configurations, we will draw on formal theories that can explain how a LoLEM might work by theorising relevant mechanisms at a level of abstraction above the specifics of the living library model. For example, at the individual (micro) and interpersonal (meso) levels these may include Intergroup Contact Theory (42, 43), Social Identity Theory (44), Social Learning Theory (45), and Personal Recovery (46). Additional theories at the organisational (macro) level may also be relevant such as the Theory of Health Empowerment (47). At this stage, and consistent with the iterative nature of realist synthesis methodology, we may conduct additional theory-driven literature searches for further formal theories that augment the theory development process. The selection and use of formal theories will be guided by the extent to which each theory helps us to further develop our programme theories.

8. Stakeholder interviews

Our second strategy to elaborate and refine our theories, will be to use realist interviewing (48) with key stakeholders. We will sample through snowballing techniques, initially drawing on the networks of our broad research team, Expert Group, and key individuals identified through our literature searching. Early interviews will inform the development of our initial programme theories, but as our theories develop, the focus of the interviews will evolve into a more specific focus on filling in gaps in our theories, and testing competing theories. Sampling will become more purposive to select interviewees with the relevant knowledge and expertise. Those willing to take part will be sent a participant information sheet and asked to consent online. Interviews will be done flexibly (to maximise participation) using individual/group interviews, online/face to face/telephone mediums, and topic guides designed to test our explanatory theories. Data will be recorded and transcribed, and coded and analysed within the theory framework in NVivo as described in steps 5 & 6.

For peer review only

9. Synthesise final theories

In the final stage of the synthesis, we will use iterative discussion within our research team, and Expert Group, to consolidate our programme theories into a final framework that articulates how a LoLEM works, for whom, and in what contexts. This will draw on data derived from literature searches, realist interviews, and from the EBCD workshops described below.

Workstream 2- Experience Based Co-Design (EBCD) of a LoLEM guide

Informed by the EBCD approach (49), we will adapt the process to meet the specific needs of this project. Key steps are outlined below.

1. Gather experiences from stakeholders to understand key issues to be addressed

In EBCD these are referred to as "touchpoints" and are identified through interviews, observations, or group discussion. In this project, we will use the data from steps 1-3 of the realist synthesis to identify key features of living libraries that need to be co-designed.

2. Set up the co-design process

Participants will be recruited through NHS Trusts, peer support charities, and existing user involvement networks across the North of England to take part in a series of monthly co-design workshops. Participants will include people with experience of using their mental health experiences to support others or to inform research and development in health services, and mental health professionals with expertise in supporting peer workers. All participants will be sent a Participant Information Sheet and asked to consent to data from the workshops being used to inform study outputs. Participants will be paid in accordance with guidance from the National Institute of Health Research (50), and offered access to individual support from the research team. Workshops will be run online to maximise engagement geographically, and through pandemic restrictions. They will be co-facilitated by an experienced trainer in peer support interventions whose work is grounded in lived expertise (KM), our PPI lead for the project (CL), and an artist (GC) with experience in co-delivering and capturing co-design processes using creative visual tools. The process will be supported by other members of the research team.

3. Run the co-design workshops

The content of each workshop will be responsive to our developing programme theories and agreed in consultation with participants during the process. The initial plan consists of four phases to the process:

a. Building rapport and trust within the group through experiences of story sharing which are fundamental to the living library model

b. Applying the experiences of story sharing to generate further understanding of the living library model, and identify barriers and facilitators to success

c. Applying the understanding of the barriers and facilitators to develop fundamental guidelines and key considerations for running a living library in a range of settings

d. Agreeing format of production of the guidelines and next steps.

4. Generate a LoLEM guide for how to set up and run a LoLEM across a range of contexts

The aim is not to write a "recipe book", but to identify key things to consider when setting up a library, and to understand how different decisions are likely to lead to different outcomes. These will be described using text and artistic impressions. Key issues anticipated include: what the library is for, where and how the library is hosted, how books are defined, recruited, trained, reimbursed, and supported; who the readers are, and how the library is offered to them; how the role of the librarian is set up, and how a LoLEM should be evaluated.

5. Celebrate and share outputs

A celebration event will be held to share the final draft of the protocol and review the process of co-design.

Patient and Public Involvement statement

Lived expertise is embedded at every level of the project. During the study design, and prior to applying for funding, we held an online consultation with four service users from our Spectrum Centre Advisory Panel at Lancaster University (51), which informed the idea of a living library (two had been living books). Perceived benefits were that this approach can be engaging, non-stigmatising and draws on a familiar and non-threatening metaphor. It has the potential to involve a wide range of books and readers due to flexibility in time/location/what is shared/ways of sharing, and the opportunity for in-depth dialogue. Challenges identified were the need for adequate training, supervision, and support for books, as well as ensuring empowerment, ownership and control by people with lived experience. Whilst designing the study, we hosted an online stakeholder event (n = 8) with services users and mental health service staff across the region. Additional considerations identified in this process included: the need to accentuate the library as additional to (rather than alternative to) PSW roles, and the need to increase diversity and access to a broad range of living books across age, ethnicity, gender, sexuality, and types of experience.

The research team includes two PPI leads with lived experience (CL & KM). They contributed to the design of the study and application for funding. They are involved in strategic management meetings and practical delivery in both workstreams, including co-facilitating

For peer review only

the EBCD workshops. They will be authors on all outputs and involved in dissemination including presentations, and as co-applicants for future grants.

Our Expert Group includes two people with lived expertise. One is a service user who has been a reader (or book) in a living library, and the other is a carer who has a lot of experience in service development at an advisory level.

Knowledge Mobilisation

Outputs from this study will include an expert informed theory of how a LoLEM could work to improve outcomes for books, and readers; and a theory informed co-designed protocol for setting up and running a LoLEM. These will be openly shared in open access journals, following RAMESES reporting guidelines (52) and through Lancaster University and Lancashire and South Cumbria NHS websites. Any amendments made to the protocol will be explained in the outcome paper on completion of the study, and updated on PROSPERO.

We also aim to have established a network of people with lived experience, and mental health support providers who are motivated to use the study outputs to establish a library. We will host a dissemination event to present the findings of the study to stakeholders including commissioners. We will invite mental health support providers, commissioners, health professional trainers, and other organisations who may benefit from hosting a LoLEM. We hope to use this to build a collaborative bid for further research funding to conduct a realist evaluation of a series of LoLEMs across different contexts.

Ethics and dissemination:

In the same way that a poorly designed LoLEM could be disempowering, stressful and even traumatizing for books, and could confirm rather than challenge existing stigma and prejudice, this could also be true for the process of co-designing the library guide. To mitigate against this, we will: conduct the study within a University (rather than NHS) context; ensure all EBCD workshops will be co-facilitated with people who have lived expertise; and ensure all participants will have access to one to one support from the research team throughout the process as required. EBCD group members will be purposively sampled to ensure a diverse range of views and minimize any power issues. We will encourage exploration of the role of a LoLEM within the broader social context of mental health support, as well as more detailed design features of the library.

All data will be provided with informed written consent and will be collected and stored securely. The detailed and personal nature of the EBCD workshop data makes it impossible to fully anonymise, so this data will not be shared openly. However, all outputs will be published in open access journals. Plain English summaries will be made available on the Lancaster University website.

For peer review only

Authors contributions

FL is the guarantor. FL led the design, funding application, and will co-lead the running of the project. PM is an Early Career Researcher who led on the ethics application, and will lead on the realist synthesis and integration of theory between the workstreams. He will co-lead the management of the study. JB advised on the search strategy. GC will co-facilitate EBCD workshops and produce artistic impressions of the developing theory. ZG, PJ, RJ, LW will help with the running of the EBCD workshops, and RJ will also screen papers and extract data in the realist synthesis. CL and KM will co-facilitate the EBCD workshops, with KM leading on co-design process, and Cl on supporting public and patient involvement. JRM will provide methodological guidance and input into the realist synthesis. SJ will be organizational lead for the EBCD workstream and consult on theory development in the realist synthesis. SF, CI, HK, SP, EM, MS have all provided strategic guidance to the design of the study. All authors contributed to and approved the final version of all protocols.

For peer review only

References

1. McManus S, Bebbington PE, Jenkins R, Brugha T. Mental health and wellbeing in England: the adult psychiatric morbidity survey 2014: NHS digital; 2016.

2. NHS Digital. Mental Health of Children and Young People in England, 2020: Wave 1 follow up to the 2017 survey October 2020 2020 [Available from: https://digital.nhs.uk/data-and-information/publications/statistical/mental-health-of-children-and-young-people-in-england/2020-wave-1-follow-up.

3. World Health Organization. World mental health report: transforming mental health for all: executive summary. World mental health report: transforming mental health for all: executive summary2022.

4. Wykes T, Bell A, Carr S, Coldham T, Gilbody S, Hotopf M, et al. Shared goals for mental health research: what, why and when for the 2020s. Journal of Mental Health. 2021:1-9.

5. Basset T, Faulkner A, Repper J, Stamou E. Lived experience leading the way: Peer support in mental health. London: Together UK. 2010.

6. Gillard S, Bremner S, Patel A, Goldsmith L, Marks J, Foster R, et al. Peer support for discharge from inpatient mental health care versus care as usual in England (ENRICH): a parallel, two-group, individually randomised controlled trial. The Lancet Psychiatry. 2022;9(2):125-36.

7. Health Education England. Supporting the individual's wellbeing, giving hope and supporting recovery 2021 [Available from: https://www.hee.nhs.uk/our-work/mental-health/new-roles-mental-health/peer-support-workers.

8. Chinman M, George P, Dougherty RH, Daniels AS, Ghose SS, Swift A, et al. Peer support services for individuals with serious mental illnesses: assessing the evidence. Psychiatric Services. 2014;65(4):429-41.

9. Lloyd-Evans B, Mayo-Wilson E, Harrison B, Istead H, Brown E, Pilling S, et al. A systematic review and meta-analysis of randomised controlled trials of peer support for people with severe mental illness. BMC Psychiatry. 2014;14(1):39.

10. The Human Library Denmark [09/11/2019]. Available from: https://humanlibrary.org/.

11. Little N, Nemutlu G, Magic J, Molnár B. Don't judge a book by its cover! The Living Library Organiser's Guide. 2011.

12. Corrigan PW, Morris SB, Michaels PJ, Rafacz JD, Rüsch N. Challenging the public stigma of mental illness: a meta-analysis of outcome studies. Psychiatric services. 2012;63(10):963-73.

13. The Reader [Available from: https://www.thereader.org.uk/.

14. Taking Part 2015/16 quarter 2 statistical release. Department for Digital, Culture, Media & Sport London: DCMS; 2015 [Available from: https://www.gov.uk/government/statistics/taking-part-201516-quarter-2-statistical-release.

15. Brewster L. The public library as therapeutic landscape: A qualitative case study. Health & Place. 2014;26:94-9.

16. Michalak EE, Morton E, Barnes SJ, Hole R, Crest.Bd, Murray G. Supporting Self-Management in Bipolar Disorder: Mixed-Methods Knowledge Translation Study. JMIR mental health. 2019;6(4):e13493.

17. Patten SB, Remillard A, Phillips L, Modgill G, Szeto AC, Kassam A, et al. Effectiveness of contact-based education for reducing mental illness-related stigma in pharmacy students. BMC medical education. 2012;12(1):1-9.

18. Chung EY-H, Tse TT-O. Effect of human library intervention on mental health literacy: a multigroup pretest-posttest study. BMC psychiatry. 2022;22(1):73.

19. Kudo K, Motohashi Y, Enomoto Y, Kataoka Y, Yajima Y. Bridging differences through dialogue: Preliminary findings of the outcomes of the human library in a university setting2011 2011.

20. Dobreski B, Huang Y. The joy of being a book: Benefits of participation in the human library. Proceedings of the Association for Information Science and Technology. 2016;53(1):1-3.

For peer review only

21. Frey C, Powell J. Building community in an academic library. Social Justice and Activism in Libraries: Essays on Diversity and Change. 2019.
22. Giesler MA. Humanizing Oppression: The Value of the Human Library Experience in Social Work Education. Journal of Social Work Education. 2021.
23. Brown D, editor School libraries as power-houses of empathy: People for loan in The Human Library. IASL Annual Conference Proceedings; 2016.
24. Maunder RD, White FA. Intergroup contact and mental health stigma: A comparative effectiveness meta-analysis. Clinical psychology review. 2019;72:101749.
25. Woods A, Hart A, Spandler H. The Recovery Narrative: Politics and Possibilities of a Genre. Culture, Medicine, and Psychiatry. 2022;46(2):221-47.
26. Yeo C, Rennick-Egglestone S, Armstrong V, Borg M, Franklin D, Klevan T, et al. Uses and Misuses of Recorded Mental Health Lived Experience Narratives in Healthcare and Community Settings: Systematic Review. Schizophrenia Bulletin. 2021;48(1):134-44.
27. Llewellyn-Beardsley J, Rennick-Egglestone S, Pollock K, Ali Y, Watson E, Franklin D, et al. 'Maybe I Shouldn't Talk': The Role of Power in the Telling of Mental Health Recovery Stories. Qualitative Health Research.0(0):10497323221118239.
28. Kwan CK. A Qualitative Inquiry into the Human Library Approach: Facilitating Social Inclusion and Promoting Recovery. International journal of environmental research and public health. 2020;17(9).
29. Skivington K, Matthews L, Simpson SA, Craig P, Baird J, Blazeby JM, et al. Framework for the development and evaluation of complex interventions: gap analysis, workshop and consultation-informed update. 2021.
30. Pawson R, Greenhalgh T, Harvey G, Walshe K. Realist synthesis: an introduction. ESRC Res Methods Program. 2004;2.
31. Bate P, Robert G. Experience-based design: from redesigning the system around the patient to co-designing services with the patient. BMJ Quality & Safety. 2006;15(5):307-10.
32. Springham N, Robert G. Experience based co-design reduces formal complaints on an acute mental health ward. BMJ Open Quality. 2015;4(1):u209153. w3970.
33. Green T, Bonner A, Teleni L, Bradford N, Purtell L, Douglas C, et al. Use and reporting of experience-based codesign studies in the healthcare setting: a systematic review. BMJ quality & safety. 2020;29(1):64-76.
34. Fylan B, Tomlinson J, Raynor DK, Silcock J. Using experience-based co-design with patients, carers and healthcare professionals to develop theory-based interventions for safer medicines use. Research in Social and Administrative Pharmacy. 2021;17(12):2127-35.
35. Shamseer L, Moher D, Clarke M, Ghersi D, Liberati A, Petticrew M, et al. Preferred reporting items for systematic review and meta-analysis protocols (PRISMA-P) 2015: elaboration and explanation. Bmj. 2015;349.
36. Pawson R. The science of evaluation: a realist manifesto: Sage; 2013.
37. Pearson M, Brand SL, Quinn C, Shaw J, Maguire M, Michie S, et al. Using realist review to inform intervention development: methodological illustration and conceptual platform for collaborative care in offender mental health. Implementation Science. 2015;10(1):134.
38. QSR International Pty Ltd. Nvivo (Version 12) 2018. Available from: https://www.qsrinternational.com/nvivo-qualitative-data-analysis-software/home?_ga=2.181156967.1157585097.1662443725-1883585101.1657638724.
39. Ouzzani M, Hammady H, Fedorowicz Z, Elmagarmid A. Rayyan—a web and mobile app for systematic reviews. Systematic Reviews. 2016;5(1):210.
40. Williams L, Rycroft-Malone J, Burton C, Edwards S, Fisher D, Hall B, et al. Improving skills and care standards in the support workforce for older people: a realist synthesis of workforce development interventions. BMJ open. 2016;6(8):e011964.

For peer review only

41. Mukumbang FC, Kabongo EM, Eastwood JG. Examining the Application of Retroductive Theorizing in Realist-Informed Studies. International Journal of Qualitative Methods. 2021;20:16094069211053516.
42. Allport G. The Nature of Prejudice. Reading, MA.: Addison-Wesley; 1954 1979.
43. Pettigrew TF. Intergroup contact theory. Annual review of psychology. 1998;49(1):65-85.
44. Tajfel H. Human groups and social categories: Studies in social psychology: CUP Archive; 1981.
45. Bandura A, Walters RH. Social learning theory: Prentice-hall Englewood Cliffs, NJ; 1977.
46. Leamy M, Bird V, Le Boutillier C, Williams J, Slade M. Conceptual framework for personal recovery in mental health: systematic review and narrative synthesis. Br J Psychiatry. 2011;199(6):445-52.
47. Shearer NBC. Relationships of contextual and relational factors to health empowerment in women. Research and theory for nursing practice. 2004;18(4):357-70.
48. Manzano A. The craft of interviewing in realist evaluation. Evaluation. 2016;22(3):342-60.
49. The Kings Fund. EBCD: Experience-based co-design toolkit. 2013.
50. NIHR. Payment guidance for researchers and professionals. 2020.
51. Spectrum Centre. Spectrum Centre - Getting Involved [Available from: https://www.lancaster.ac.uk/health-and-medicine/research/spectrum/research/getting-involved/#d.en.405692.
52. Wong G, Greenhalgh T, Westhorp G, Buckingham J, Pawson R. RAMESES publication standards: realist syntheses. BMC medicine. 2013;11(1):21.

For peer review only

Figure 1. Library of Lived Experience for Mental Health (LoLEM) Study Design

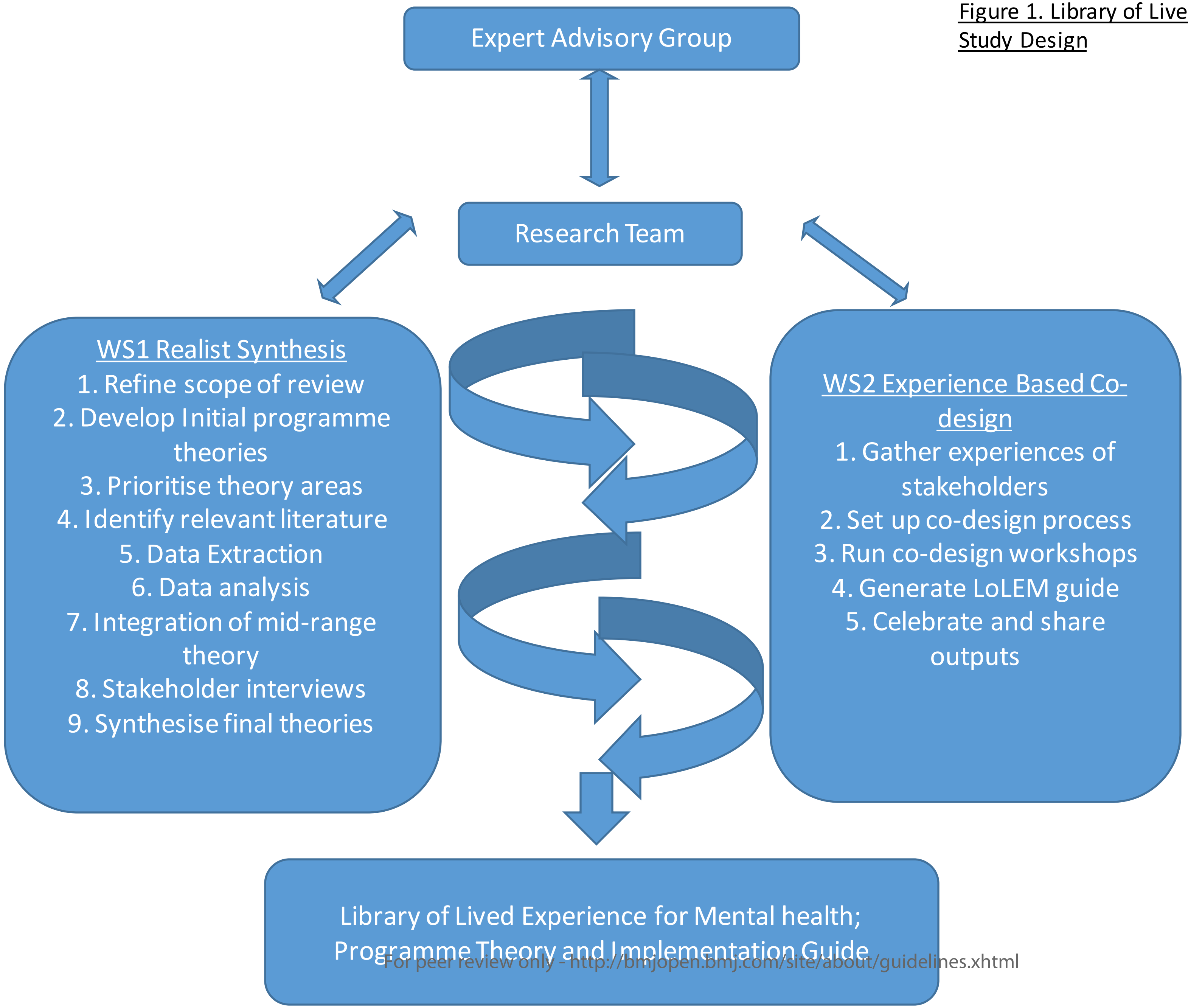

For peer review only - http://bmjopen.bmj.com/site/about/guidelines.xhtml
